# The STRidER Report on Two Years of Quality Control of Autosomal STR Population Datasets

**DOI:** 10.3390/genes11080901

**Published:** 2020-08-07

**Authors:** Martin Bodner, Walther Parson

**Affiliations:** 1 Institute of Legal Medicine, Medical University of Innsbruck, 6020 Innsbruck, Austria; 2 Forensic Science Program, The Pennsylvania State University, University Park, PA 16801, USA; walther.parson@i-med.ac.at

**Keywords:** database, STR profile, population data, error, allele frequency, genotype

## Abstract

STRidER, the STRs for Identity ENFSI Reference Database, is a curated, freely publicly available online allele frequency database, quality control (QC) and software platform for autosomal Short Tandem Repeats (STRs) developed under the endorsement of the International Society for Forensic Genetics. Continuous updates comprise additional STR loci and populations in the frequency database and many further STR-related aspects. One significant innovation is the autosomal STR data QC provided prior to publication of datasets. Such scrutiny was lacking previously, leaving QC to authors, reviewers and editors, which led to an unacceptably high error rate in scientific papers. The results from scrutinizing 184 STR datasets containing >177,000 individual genotypes submitted in the first two years of STRidER QC since 2017 revealed that about two-thirds of the STR datasets were either being withdrawn by the authors after initial feedback or rejected based on a conservative error rate. Almost no error-free submissions were received, which clearly shows that centralized QC and data curation are essential to maintain the high-quality standard required in forensic genetics. While many errors had minor impact on the resulting allele frequencies, multiple error categories were commonly found within single datasets. Several datasets contained serious flaws. We discuss the factors that caused the errors to draw the attention to redundant pitfalls and thus contribute to better quality of autosomal STR datasets and allele frequency reports.

## 1. Introduction

Data accuracy is fundamental in forensic genetics. Quality considerations cover wide-ranging aspects of sampling, data generation, standardization, validation, interpretation, storage, data handling, proficiency testing, laboratory management and accreditation [1,2,3,4,5,6,7,8,9,10]; for a list of recent guidance documents, see [11]. Efforts have to encompass the generation of DNA profiles from crime scene samples and reference persons as well as those generated for population databases [12,13,14,15]; the allele and haplotype frequencies stored in the latter are crucial for statistical evaluation in forensic casework, identity testing and kinship investigations. Earlier studies demonstrated the importance of quality control (QC) in haploid DNA marker data generation and databasing [16,17] and ultimately led to the establishment of the two legally valid forensic databases EMPOP [12] and YHRD [13] for mitochondrial DNA (mtDNA) and Y-chromosomal haplotypes, respectively, more than two decades ago. Two forensic genetic journals, *Forensic Science International: Genetics* (*FSI: Genetics*) and the *International Journal of Legal Medicine*, made QC via EMPOP and YHRD mandatory for submissions containing respective frequency data [17,18,19]. Autosomal short tandem repeats (STR) are the most widely applied forensic DNA markers. Despite the existence of (national) databases since 1995, broad discussion of the ethical and legal framework [20] and dataset cleaning steps at the population level [21,22,23], autosomal STR allele frequency data have not been subject to any such compulsory, publicly presented, pre-publishing QC until recently. Errors and inconsistencies observed in published allele frequency tables [24,25,26] ultimately led to conceiving a QC platform also for autosomal STR data under the umbrella of the International Society for Forensic Genetics (ISFG) to minimize such mistakes. Building upon the previously established European Network of Forensic Science Institutes (ENFSI) DNA Working Group autosomal STR Population Database (STRbASE) [27], the project was implemented as the STRs for identity ENFSI Reference database (STRidER), available online at https://strider.online. STRidER was launched in 2017 with the intention to create a freely accessible functional forensic online platform for databasing, rarity estimation, interpretation and QC of autosomal STR data [14]. STRidER acts independent of any journal and publication intentions and has been endorsed by the ISFG in appreciation of its efforts. This article reports insights from the first two years of STRidER QC on autosomal STR population datasets and constitutes the largest systematic review of such data at the level of individual genotypes so far available to the forensic genetic community. 

## 2. Autosomal STR Data Quality Control on STRidER

### 2.1. What STRidER Quality Control Is (Not): Rationale and Workflow

It is an integral part of the responsibility of authors, reviewers, editors and database curators to assure utmost correctness of autosomal STR, or any, datasets before their publication or upload in accordance with good scientific practice guidelines. To protect genetic privacy, only allele frequency tables have been reported in scientific publications of STR population data and genotypes were not requested during peer-review of the accompanying article. This, however, did not prevent mistakes from being caught in the limited data assessable [14,24,26]. Occasionally, only allele frequency bar charts were published, preventing any further QC attempts [28]. 

To initiate autosomal STR dataset QC at STRidER, authors are requested to submit the complete and unshuffled genotype tables for capillary electrophoresis (CE) data, while FASTA-like nucleotide string files are required for massively parallel sequencing (MPS) data. Backward compatibility of MPS results with the existing CE-based data is of major importance [29]. To assess difficulties in conversion from a practical perspective, STRidER currently requests “translated” MPS STR alleles mimicking length-based CE results. Independently generated CE data are not required for MPS datasets, but might be requested to resolve ambiguities. STRidER accepts datasets from diverse worldwide populations and forensically relevant autosomal STR markers that typically consist of 100–1000 genotypes. Larger STR datasets may reveal additional alleles; however, they are expected to be rare [30,31] and not statistically relevant for a correspondingly decreasing minimum allele frequency bound such as 5/2n that has been applied in forensic genetics over decades [32] and is used in STRidER. Accompanying information on the population of origin, sampling strategy, methodology, kits, analytical settings applied and ethical clearance has to be provided. Complete raw data, reflecting the laboratory process, need to be retained and are specifically requested during inspection. A suite of in-house software tools is applied to scrutinize data in a stepwise process, in a procedure optimized for the detection of common data idiosyncrasies and conspicuities. STRidER QC aims to assess the plausibility and quality of submitted datasets *a posteriori* and cannot be regarded as a comprehensive independent evaluation of all raw data. Submitters are invited to comment on all findings. A unique, permanent and traceable STRidER accession number is assigned to the dataset and provided to the data submitters for reference after positive evaluation. This number also serves as an indicator of successful STRidER QC for journal editors, reviewers and readers. STRidER aids the reviewing process, while the final assessment of eligibility for publication remains with the editors and reviewers who need to consider additional information and the existing body of literature in their evaluation. STRidER provides allele frequencies calculated from datasets that successfully passed QC to the authors and includes them in regularly disseminated releases of the growing online database. The submission and QC processes are amended as required [14,17]. Updates to STRidER are announced in a newsletter (https://strider.online).

### 2.2. STRidER Quality Control Submissions in the First Two Years

**Figure 1:**
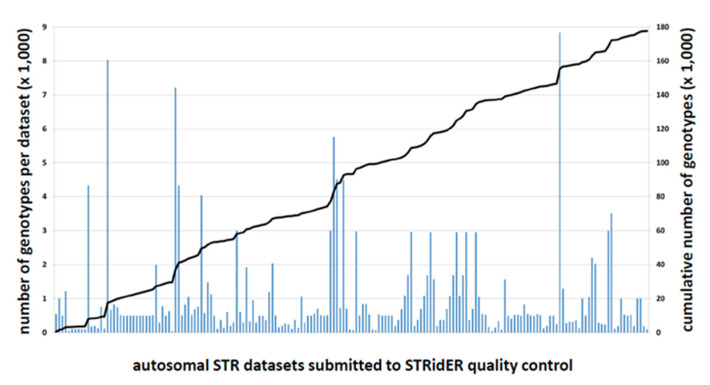
Submissions to STRidER (STRs for Identity ENFSI Reference Database) for quality control in the first two years. Bars indicate the 184 submitted datasets in chronological order of submission between July 2017 and July 2019, dataset size according to the left axis; line indicates the cumulated number of samples over all submitted datasets according to the right axis.

STR data submissions commenced on 17 July 2017 after the publication of “Revised guidelines for the publication of genetic population data” in *FSI: Genetics* [17] on 22 June 2017 that made autosomal STR data QC via STRidER mandatory. In the first two years, 184 datasets originating from 36 countries of Africa, the Americas, Asia and Europe were sent to STRidER for QC (Table S1 and online map at https://bit.ly/3fiSSJC). They were almost exclusively intended for publication. The total number of submitted genotypes was 177,595. Nineteen of the 184 datasets (10.3%) contained sequenced STR alleles (in total 3886 genotypes; mean: 205; median: 140 per dataset). They were generated using kits from Verogen (San Diego, CA, USA; *n* = 9; 47.3%), Promega (Madison, WI, USA; *n* = 7; 36.8%), Thermo Fisher Scientific (Waltham, MA, USA; *n* = 2; 10.5%) and IPE Biotechnology (Beijing, China; *n* = 1; 5.2%). The remaining 165 datasets (89.7%) were compiled from CE data (in total 173,709 genotypes; mean: 1053; median: 522 per dataset) (Figure 1, Table 1). Some submissions consisted of several distinct datasets (e.g., population groups from the same country). Resubmissions of datasets after (multiple) withdrawal or rejection cycles were subjected to all QC measures once again and counted autonomously.

## 3. Results of STRidER Quality Control

Results portrayed in this report reflect the status six months after the initial two-year period. Statistics including datasets pending at the time of writing may thus change over time.

### 3.1. Overall Outcome

Of the 184 datasets (112 novel and 72 revised) submitted to STRidER in the first two years, 48 (26.1%) successfully passed QC. Forty-one datasets (22.3%) were withdrawn by the submitters at different stages during the process, mostly after STRidER inquiries. When reasons were specified, they included wide-ranging quality problems and anticipated rejection due to nonconformity with ISFG recommendations [14] or *FSI: Genetics* guidelines [17]. STRidER rejected 58 datasets (31.5%) for quality concerns according to criteria specified in more detail below. For the datasets with completed QC (*n* = 147), the acceptance rate was 32.7%, while 39.5% were rejected and 27.9% were withdrawn. For a further 37 datasets (20.1%), evaluation is currently pending, while STRidER is awaiting replies on QC inquiries. It is anticipated that in many cases a lack of response over many months equals withdrawal (Table 1).

### 3.2. Error Statistics

Statistics were built from errors confirmed at the time of writing, regardless of the progress of QC for the individual dataset. Hence, they are not necessarily exhaustive—withdrawn and rejected datasets might harbor additional errors to those that led to the termination of QC, and pending datasets might contain more errors than those already noted. *Vice versa*, resubmitted datasets were mostly skewed toward lower error rates. In any event, the error rate revealed by independent repetition of analyses or complete raw data evaluation is expected to be higher than that, which can be detected in *a posteriori* checks. Obvious error reasons identified and confirmed after specific investigation are discussed along with the error types, with the intention to mitigate their future occurrence. Error categories were arranged as they have proven useful in two years of hands-on QC work, and might be combined or split in further ways. Some errors may possibly be assigned to more than one class. Error prevalence was counted as occurrence per dataset and is given as a “hit list” of error types. This does not allow inferring a general proportion of affected genotypes within the respective datasets. Such quantitative measures are given where meaningful. They can be biased for the reasons outlined above and are not always straightforward in calculation; some mistakes might cause a disproportional number of errors [33]. For example, two completely different situations might cause all genotypes in a data table to be wrong—a single erroneous STR locus name or a high number of individual errors.

#### 3.2.1. CE Dataset Error Charts

Of the 165 submitted CE datasets, seven (4.2%) passed QC without revealing any detectable errors; six thereof were, however, ameliorated resubmissions where errors previously pinpointed by STRidER had been corrected. The remaining 158 CE datasets were affected by up to six of the error types listed below (Table 2).

(i) *Identical Genotypes*

Multiple copies of genotypes were found in 63 (38.2%) of the submitted datasets, both within and across datasets. Up to 106 non-unique genotypes occurred per dataset at a copy number ranging between two and six. Almost always, their identifiers were different. Identical genotypes (over >15 loci) indicate sampling or data compilation mistakes leading to the repeated inclusion of one individual or homozygous siblings, and may also derive from positive control samples used in multiple experiments.

(ii) *Non-Ascending Allele Pairs*

Fifty-eight datasets (35.2%) contained up to 38 non-ascending allele pairs. Deviations from the convention of reporting alleles at a given locus in ascending manner (e.g., incorrect *12|6* instead of *6|12*) were almost exclusively shown to indicate errors beyond the wrong order after raw data inspection; e.g., *12|6* could derive from a manual transcription (so-called clerical) error of the true genotype *12|16*. This error type also affected genotype-based parameters and masked identical genotypes. Non-ascending allele pairs are considered to derive mostly from transcription errors or column shifts in table preparation.

(iii) *Errors in Allele Nomenclature*

STR allele designations not conforming to forensic nomenclature were found in 29 (17.6%) datasets on up to 30 occasions. Calls included a plethora of impossible (e.g., *9+*, *−9*, *18.9*) and highly implausible (e.g., *0*, *99*, *115*, *1314*) repeat numbers, letters (*9R*, *13NEW*, *M*, *OL*, *R*, *X*, *Y*) as well as commas delimiting incomplete repeat units (e.g., *9,3*) mixed with the common decimal points (e.g., *9.3*); “*1*” and “*99*” designations were accepted only when representing rare alleles outside the established allele categories. Impossible amelogenin alleles (*M*, *Y2X*, numbers) and genotypes (*Y|Y*) were reported. These errors may derive from in-house or software placeholders for manually scrutinized, homozygous, silent, missing or off-ladder alleles, non-separated cells, typos or column shifts in table preparation.

(iv) *Erroneous Allele Calls*

Alleles close to or within the regular length range, yet unobserved or unexpected for the given locus and population, turned out to be erroneous after raw data inspection in 14 (8.5%) datasets on up to 34 occasions. Such allele calls derived from transcription errors and incorrect manual extrapolation of intermediate allele length. Additional alleles were found to be wrong after random raw data inspection in two of the abovementioned and three further datasets, raising their total number to 17 (10.3%).

(v) *Incomplete Genotypes*

Up to 64 incomplete profiles with missing allele pairs or *0|0* genotypes at up to nine interspersed loci were found in 16 (9.7%) datasets. Only complete genotypes are accepted for STRidER databasing, since population samples intended for allele frequency databases should consist of reference material with predictably good quality [1,14]. Additional error was introduced when the number of sampled alleles was not adapted in the calculation of genetic parameters for the affected loci.

(vi) *Errors in Locus Nomenclature*

Incorrect locus names were reported in 10 (6.1%) submissions. Locus names were completely missing in one dataset, the remaining errors comprised incomplete (e.g., *D6*, *GATAB05*) and obviously misspelled (e.g., *D21S12*, *SEE33*, *TH02*) designations. It is likely that dragging errors in table preparation contributed to the latter. Even minimal nomenclature errors might lead to confusion with (upcoming) other STR loci, such as *D2S441* vs. *D2S411* [34,35]. In one submission, locus D5S2500 was incorrectly assigned by the typing kit manufacturer instead of D5S2800 [36]. Unequivocal variation, such as *D22-GATAB04* vs. *D22GATAB04*, *HUMVWA* vs. *vWA* vs. *VWA* and *PentaD* vs. *Penta D*, was not considered as an erroneous marker name. 

(vii) *Aneuploidy*

Nine datasets (5.5%) revealed non-diploid allele numbers including genotypes with single alleles, deriving from erroneous reporting of homozygosity or loss of the second, correctly called, allele during table preparation, as well as tri-allelic loci (that are not considered for allele frequency databasing [14]). Consistently tetra- or haploid loci were also reported.

(viii) *“QC killers”: Shuffled Genotypes, Lack of Raw Data*

During submission, contributors warrant the availability of complete raw data and the submission of original genotypes. A dataset with shuffled genotypes or missing raw data cannot be properly assessed [14,22]. Nine datasets (5.5%) turned out not to comply with these declarations during QC and had to be rejected, independent of correctness.

(ix) *Non-Unique Sample Identifiers*

Identical sample identifiers listed for multiple different genotypes occurred in seven (4.2%) datasets as one or two pairs.

(x) *Information Mismatch*

Mismatches between the provided non-genetic information and the actual submission occurred in five (3.0%) datasets, including deviations in expected sample size and STR loci. They pinpointed errors and omissions in data compilation. Missing loci already reported elsewhere were not considered.

(xi) *Locus Swapping*

A complete transposition of alleles between two loci was detected in three datasets (1.8%).

(xii) *Loss of Intermediate Alleles*

In two (1.2%) datasets, the additional bases of intermediate STR alleles were partly lost by erroneously rounding the “decimal places” to full repeat numbers (e.g., *9.3* reported as *9*). This was caused by incorrect settings in the applied software.

#### 3.2.2. MPS Dataset Errors

Errors encountered in MPS datasets were generally rarer than in CE data submissions, but this may simply be a reflection of the small number of datasets (*n* = 19), produced by early-adopting laboratories. Findings included some of the abovementioned categories: identical genotypes, incomplete genotypes, erroneous locus names, allele transpositions between loci and non-uniform sample identifiers over loci of the dataset. Further error types specific to sequence data were encountered:

(i) *Erroneous Sequences*

Due to text handling errors during file preparation, some allele sequences did not match the locus reference sequence or its expected variation described in the *STRSeq* catalog [37] and the Forensic STR Sequence Structure Guide [38] available from STRidER at https://strider.online/nomenclature.

(ii) *Inconsistencies in Sequence Orientation*

According to the ISFG considerations on minimal nomenclature requirements in MPS of forensic STRs [29], all sequences should be reported in forward (5′−3′) direction that is further described in [38]. Loci reported in reverse direction resulted from non-compliant versions of the software.

(iii) *Errors in CE Translation*

STRidER identified counting errors in the number of repeat units revealed by sequencing and beyond QC, pinpointed expected discrepancies between apparent CE results and sequence-based counting caused by flanking region (FR) indels in some samples.

The difficulty of retrieving MPS allele sequence data including sufficient FR information without advanced bioinformatics knowledge has in some cases hampered QC. FR output between unambiguous coordinates has been postulated [39]. STR sequence nomenclature was not assessed during QC; the highly desired forensic recommendation is currently under development [29,37,38,39,40]. Further errors detected in CE-translated allele tables were similar to those described in the previous chapter, but not taken into account for a decision on the MPS datasets. However, discrepancies between the submitted allele sequences and the CE conversion tables often pinpointed errors in the MPS data.

## 4. Discussion

### 4.1. Origin and Effect of Errors in Autosomal STR Datasets

With the evolution of chemistry, equipment and software, the generation of autosomal STR datasets has become largely automated once suitable DNA extracts are available in sufficient number and quality. Publication of the resulting allele frequencies requires a comparably small workload and manuscript templates for data announcement have even been provided [19,41]. It can be assumed that the sheer obligation of external QC via STRidER [17] has caused higher diligence in data preparation or migration of manuscripts to other journals. Maintaining the highest standards is essential for good scientific practice and has even been called a “moral obligation” within the legal system [33]. With respect to that, the degree of laxness of forensic laboratories in assuring population data quality revealed by STRidER (Table 2), with a withdrawal and rejection rate constantly fluctuating around two-thirds during and after the first two years (Table 1), was completely unexpected. Some datasets contained errors, which would have had a major impact on STR allele frequencies and forensic genetic parameters and thus seriously affect the veracity of probabilistic calculations. Many of the remaining errors detected in the submitted datasets might be subsumed under flaws of *per se* almost negligible effect. In a large dataset, the impact on STR allele frequencies would be minor because of their rarity. Small frequency differences in some of the loci have been described as non-problematic for calculations when many loci are used [25,42,43,44]. Still, they can be relevant: the failure to report rare alleles would not easily be detected [33] and likely be “negligible” in frequency calculations when minimal allele frequencies are applied, but their abundance can be useful for predicting unknown genetic variation in a population—cf. the singleton fraction ĸ considered in haploid marker population samples [45]. In any case, often an accumulation of multiple errors and error types was found per dataset. During STRidER QC of 184 STR datasets, even “small” flaws frequently indicated general problems with data handling and quality in the submitting institution. This link would also explain the high number of retracted datasets and long-time pending, often minor, STRidER QC inquiries (Table 1). Thus, the extent, diversity and dispersal of apparently small errors in a dataset are highly informative for *a posteriori* QC that can only pinpoint the obvious. 

In an extreme interpretation, it could be argued that almost all errors in submissions occurred solely out of the necessity of providing specific file formats for STRidER. Unfamiliar formats can cause errors that do not occur in regular reports from the same laboratory [33,46] and the role of spreadsheet software in the introduction of errors has been discussed (e.g., [47]). However, errors in datasets published independent of STRidER do not support this as a singular explanation (see Section 4.2) and the most common and probably most serious error—identical genotypes—clearly is independent of STRidER submission formats. It may further be assumed that the most obvious fails would not have made it into publication [48] even without STRidER QC; again, published datasets containing obvious table errors or allele frequencies that do not sum up to 1 suggest the contrary [14,24,26] and identical genotypes cannot be detected from frequency data inspection. In any event, it has to be taken into account that novel errors might also be introduced in the publication process after any QC, e.g., during the setting of tables, and not be detected during proofreading.

Importantly, errors were not caused by shortcomings intrinsic to the typing methodologies. Errors were human-based and introduced during sampling, manual data analysis and management, reporting and transfer. In fact, the most common error types found in the submitted datasets can be explained by negligence either in sample set and data compilation and/or during manual transcription of results (clerical errors), as outlined above (Table 2). Anonymity of samples without the “casework option” of double-checking with or correlation to other results, the high complexity introduced by the high number of samples and the lack of control mechanisms, such as double reading of results or confirmatory analysis of samples by another laboratory, have been considered contributory factors [16]. Resubmitted dataset versions were of generally higher quality than fully novel submissions to STRidER even by the same authors, confirming the major human component in the emergence of errors. No improvement of data quality in novel submissions was noted in the course of time. 

### 4.2. Literature Reports on Errors in Forensic Autosomal STR Data

The creation and quality assurance of (in-house) forensic allele frequency databases have a long tradition of discussion, e.g., [20,30,31,32,49,50,51,52]. Limitations by lacking QC on population datasets have been exemplified [15,26,53], but investigations on autosomal STR typing error and its prevention have generally focused on casework. In this respect, collaborative exercises and proficiency tests among laboratories allow monitoring forensic expertise and grant valuable insight into the quality of typing and accuracy of results. Such trials predate the analysis of STR markers and are regularly organized with varying focus by diverse entities in the forensic field, such as CTS [54], EDNAP [55], ESWG-ISFG [46], GEDNAP [48], GeFi [28], GHEP-ISFG [56] and others. Typically, aliquots or identical replicates of a few samples or stains are sent to participating laboratories for analysis; results are reported back to the organizers and (blindly) evaluated, sometimes also considering raw data to investigate the origin of inconsistencies in more detail [57]. Format, design, parameters, aims and complexity of such inter-laboratory exercises differ from regular circumstances in the respective laboratories. The expected genetic profiles are known and discordances in raw data and/or reported results can be directly identified. Therefore, performance and error rates do not mirror results from routine casework analyses [3,33,58,59] that have also been investigated [60]. They cannot be used to extrapolate results from population studies; hence, direct comparability to STRidER QC results is limited. Unsurprisingly, error rating can be non-trivial in collaborative exercises [33] and is not uniform among reports [60]; for example, counting of erroneous proportions is performed for samples (profiles), loci (genotypes) or alleles of a dataset. Still, findings indicate typical problems in autosomal STR typing and reporting that are also encountered during STRidER QC, and are worth a discussion here.

The stated magnitude of errors or discordance in autosomal STR results rarely exceeded 1%, with a maximum of 4%, both in mock casework trials and kinship exercises, e.g., [33,46,54,61,62,63,64,65,66,67,68,69,70,71,72,73]. Higher error rates in mixture studies including low-template components [74,75] were not considered. Regardless of the method used in testing, human clerical errors in transcription of results were persistently reported, e.g., [21,33,46,48,54,64,66,69,74,75,76,77]. While most authors did not detail the type of human error, a broad spectrum mirrored by the STRidER QC can be expected. This is supported by more detailed proficiency test reports that specified manual allele calling errors, lacking separators between the two alleles at one locus, exchange of alleles between loci [75] and the tendency toward the concentration of errors in some “not so good” submissions [22,33,61,64,65,75]. Further analyses on (published) datasets confirmed the role of transcriptional errors [22] and found duplicate genotypes across database subsets [21,22,78], locus name misspelling [34], as well as allele swapping [26]. Information on errors and error rates in MPS STR typing is expected from ongoing collaborative exercises (P.A. Barrio et al., under review). Valuable insights for comparison to STRidER QC results were yielded from complete genotype-based re-evaluations of entire STR population datasets. An erratum [25] reported on the retyping of the Federal Bureau of Investigation (FBI) dataset (*n* ≈ 1175) [79]. Irrespective of corrections owing to technological limitations of the time, errors comprised “human error”, typically due to manual data handling, and “data or sample processing errors”, such as genotype duplications. Genotyping errors were revealed in 27 samples (2.3%) and required a frequency correction for 51 alleles with a maximum change of 1.8% [25]. A re-analysis of the National Institute of Standards and Technology (NIST) dataset (*n* = 1036) [80] was performed using MPS [44]. Beyond effects of methodological advances and changes in reporting, the corrigendum reported “laboratory and data analysis errors” affecting 12 samples (1.2%) at 37 loci. The maximum change in allele frequency was 1.0% in one subpopulation [44]. In both datasets, the total sample numbers for some markers changed after re-analysis, affecting further genetic parameters. The discrepancy in random match probability results was estimated to be less than 2-fold in both the FBI and the NIST datasets. A review of another NIST dataset (*n* = 700) [81] revealed erroneous alleles in six samples (0.9%) (available at https://strbase.nist.gov/NISTpop.htm). A large ENFSI study that compared genotyping results from 26 populations applying multiple CE kits in parallel found that less than 0.06% of reported results were discordant between kits caused by clerical errors. Duplicate genotypes were also revealed [22].

Precautions to minimize clerical STR errors have been described [6,82,83]. It has been reported that the advancement of methodology and standardization in STR typing have led to a reduction of technical errors [33,46], while the anticipated avoidance of human error [76] has not yet occurred. 

Human error is a known factor also in STR intelligence databasing and casework [8,9,60]. Furthermore, the high risk of manual transcription has been described for haploid marker data [18]. A pivotal collaborative exercise on mtDNA Sanger-type sequencing [16] reported insights similar to this report, helped to identify sources of errors and ultimately promoted the introduction of QC mechanisms in the field. A haplotype error rate of 10.7% was revealed. Errors relevant and comparable to autosomal STR typing were mostly clerical errors, as well as sample mix-ups, nomenclature discrepancies and artificial recombination (mix-up of the sequenced regions) paralleling shuffled genotypes in STRidER submissions.

### 4.3. The Evolution of STRidER Quality Control in Its First Two Years

STRidER is a service that is offered to the scientific community free of charge. The QC process evolved from a laborious and communication-intensive hands-on guided tour for submitters through each dataset to a more automated procedure enabling STRidER to handle the overwhelming amount of submitted datasets (Figure 1). This development was empowered by a steep learning curve regarding the nature, occurrence, frequency and detectability of expected [14] as well as unexpected errors. It was recognized that most submitters corrected pinpointed errors (e.g., “*please check TPOX alleles 12|9 in sample 6*”), but did not diligently re-inspect their entire dataset, while still stating this fact—until the next error was addressed. Therefore, STRidER moved onto more general comments (e.g., “*please check allele order*”), encouraging submitters to assume their responsibility of good scientific practice in providing high quality data, with the aim of also recognizing errors beyond what an *a posteriori* QC tool can reveal. STRidER QC is intended as a QC platform and not a teaching tool; therefore, rejection of erroneous datasets is inevitable. Thresholds for rejection were discussed in the framework of the EU-funded dna.bases project and established as follows: lack of raw data, shuffled genotypes, presence of more than seven errors, more than five errors of one category, more than five total errors from three or more categories or errors from more than three categories. Based on own and published (see above) observations of error concentration, these thresholds were applied independent of the actual dataset size. To speed up submission, an online submission tool has been implemented at https://strider.online/qc. It also covers all meta-information necessary for QC and is already effective for CE datasets. The tool assures data completeness and gives immediate feedback on typical issues that need to be resolved before upload. While STRidER is still able to monitor the entire process, automation has curtailed the detection of some basic errors originally contained in datasets that are now corrected before successful submission.

### 4.4. Reactions on STRidER Quality Control

A number of submitters did not take STRidER’s efforts in their stride and expressed reluctance to conform to its necessities even when implausibility was specifically pinpointed. The *laissez-faire* approach, manifested also by uncorrected, or even worsened, re-submissions delayed the QC process. In the same way, a study that revealed erroneous published STR allele frequency data received “helpful answers” from only half of the authors [26]. On the contrary, the majority of feedback on STRidER QC conveyed appreciation. Authors declared better training of staff to increase awareness about data quality. With appropriate control mechanisms in place, clerical errors are less likely to occur [16]. Encouraging feedback from self-inspection of five datasets (2.7%) after submission to the STRidER QC pipeline mirrors this attention—inspired by STRidER inquiries, corrections to genotypes plausible enough to pass QC were requested. Recently, QC in analogy to STRidER has also been claimed for X-chromosomal STR datasets [84].

## 5. Conclusions

Autosomal STR population datasets have not yet been exposed to such detailed QC, validation and confirmation as illustrated in this report. The importance of STRidER acting as a quality filter prior to the publication of autosomal STR allele frequency data became evident from the results of the first two years of STRidER QC. The remarkably large proportion of mostly clerical errors in datasets submitted to STRidER (Table 2) and affirmed by literature reports explicitly includes the chance for improvement in data quality, once more diligence and scrutiny are applied. In the meantime, freely available tools that aid autosomal STR dataset examination have been presented [15]. The unique insights described here are anticipated to raise awareness about quality in the most widely applied forensic genetic marker to the benefit of the entire forensic community. It will remain this community’s full responsibility to assure that only utmost correct data are applied, as contributed by the continuously growing STRidER autosomal STR allele frequency database that is supported by worldwide submissions.

## Figures and Tables

**Table 1:** Statistics of autosomal short tandem repeat (STR) datasets submitted to STRidER in its first two years and quality control outcome.

	All	CE ^1^	MPS ^2^
	Number of Genotypes		
Total	177,595	173,709	3886
Mean per dataset	965	1053	205
Median per dataset	506	522	140
	**Number of Datasets**		
Total	184	165	19
Passed QC	48	35	13
Withdrawal during QC	41	36	5
Rejection by STRidER	58	58	0
QC pending	37	36	1
	**Proportions of Datasets with completed QC (%)**		
Acceptance rate	32.7	27.1	72.2
Withdrawal/Rejection rate	67.3	72.9	27.8

^1^ Length-based genotypes generated by capillary electrophoresis (CE). ^2^ Genotypes generated by massively parallel sequencing (MPS). Note: Results as per six months after the initial two-year period. QC, quality control.

**Table 2:** Statistics of errors found in the 165 autosomal STR datasets generated by capillary electrophoresis (CE) and submitted to STRidER in its first two years.

			*n*	(%)
**Datasets Revealing Errors**			**158**	**95.8**
**Error categories**	(i)	Identical genotypes	63	38.2
	(ii)	Non-ascending allele pairs	58	35.2
	(iii)	Allele nomenclature errors	29	17.6
	(iv)	Allele calling errors	17	10.3
	(v)	Incomplete genotypes	16	9.7
	(vi)	Errors in locus nomenclature	10	6.1
	(vii)	Aneuploidy	9	5.5
	(viii)	No raw data/shuffled data	9	5.5
	(ix)	Identical identifiers	7	4.2
	(x)	Information mismatch	5	3.0
	(xi)	Locus swapping	3	1.8
	(xii)	Loss of intermediate alleles	2	1.2
**Datasets Revealing No Errors**			**7**	**4.2**

Note: Sum of datasets allocated to categories is larger than 100% because of datasets that harbored multiple error categories. Results as per six months after the initial two-year period.

## Supplementary Materials

The following are available online at https://www.mdpi.com/2073-4425/11/8/901/s1, Table S1: Geographic origin of the 184 autosomal STR datasets submitted to STRidER in its first two years.
