# Supplementary material for: The STRidER Report on Two Years of Quality Control of Autosomal STR Population Datasets
Source: Genes (Basel). 2020 Aug 7;11(8):901. doi: 10.3390/genes11080901 (PMC7463946; doi:10.3390/genes11080901)
Supplement: Supplementary file 1 [file genes-11-00901-s001.pdf]

The STRidER report on two years of quality control of autosomal STR population datasets

Martin Bodner, Walther Parson

Table S1: Geographic origin of the 184 autosomal STR datasets submitted to STRidER in its first two years (online map available at https://bit.ly/3fiSSJC)

continents	countries	datasets			
		number	[%]	number	[%]
**AFRICA**	Algeria, Egypt, Libya, Morocco*	1	0.5	10	5.4
	Ghana	2	1.1		
	Mauritius	2	1.1		
	Mozambique	1	0.5		
	Cameroon, Chad, Niger, Nigeria, Senegal*	3	1.6		
	Sierra Leone	1	0.5		
**AMERICAS**	Argentina	28	15.2	45	24.5
	Brazil	6	3.3		
	Mexico	2	1.1		
	Paraguay	4	2.2		
	USA	5	2.7		
**ASIA**	China	81	44.0	113	61.4
	India	7	3.8		
	Iraq	3	1.6		
	Malaysia	4	2.2		
	Pakistan	2	1.1		
	Philippines	2	1.1		
	Saudi Arabia	3	1.6		
	Thailand	9	4.9		
	Vietnam	2	1.1		
**EUROPE**	Austria	1	0.5	13	7.1
	Italy	2	1.1		
	Poland	1	0.5		
	Switzerland	1	0.5		
	Slovenia	2	1.1		
	Spain	3	1.6		
	Sweden	1	0.5		
	UK	1	0.5		
	Ukraine	1	0.5		
**UNKNOWN**	unspecified African origin	1	0.5	3	1.6
	unspecified Asian origin	1	0.5		
	unspecified "Caucasian" origin	1	0.5		

*mixed datasets
